# Methylation Markers for Urine-Based Detection of Bladder Cancer: The Next Generation of Urinary Markers for Diagnosis and Surveillance of Bladder Cancer

**DOI:** 10.1155/2012/503271

**Published:** 2012-06-18

**Authors:** Thomas Reinert

**Affiliations:** Department of Molecular Medicine, Aarhus University Hospital, 8200 Aarhus N, Denmark

## Abstract

Cancer of the urinary bladder is the fifth most common neoplasm in the industrialized countries. Diagnosis and surveillance are dependent on invasive evaluation with cystoscopy and to some degree cytology as an adjunct analysis. Nomuscle invasive bladder cancer is characterized by frequent recurrences after resection, and up to 30% will develop an aggressive phenotype. The journey towards a noninvasive test for diagnosing bladder cancer, in order to replace or extend time between cystoscopy, has been ongoing for more than a decade. However, only a handful of tests that aid in clinical decision making are commercially available. Recent reports of DNA methylation in urine specimens highlight a possible clinical use of this marker type, as high sensitivities and specificities have been shown. This paper will focus on the currently available markers NMP22, ImmunoCyt, and UroVysion as well as novel DNA methylation markers for diagnosis and surveillance of bladder cancer.

## 1. Introduction

Cancer of the urinary bladder is the fifth most common neoplasm in the industrialized countries and in the Unites States, with an estimated 70,530 new cases of bladder cancer diagnosed and with 14,680 deaths in 2010 [1]. Risk factors associated with the development of bladder cancer are mainly smoking and to a lesser extent workplace exposure to carcinogens [2, 3]. No genomic risk markers have been discovered apart from a few SNPs with a very low increase in relative risk [4].

 In approximately 70% of all cases the patients will present with nonmuscle invasive bladder cancer (NMIBC) of stages Ta, T1, or Tis, whereas the remaining 30% of the tumors will be muscle invasive stage T2–4 bladder cancers (MIBC). Tumor recurrences are frequent (70%) in patients with NMIBC, whereas progression to MIBC is less frequently observed (10%–30%) [5]. The standard treatment of NMIBC is transurethral resection (TUR) complemented by use of intravesical immunotherapy or chemotherapy in order to preclude recurrence and progression [6]. Risk factors associated with recurrence are tumor size, multiplicity, stage, and grade, whereas the risk factors for progression are tumor size, multiplicity, stage, high grade, and the presence of carcinoma in situ (CIS) [7]. The sensitivity of cystoscopy for NMIBC is close to 80% for white light cystoscopy and 96% when using hexaminolevulinate (HAL). The sensitivity of white light cystoscopy decreases to 48% and 68% for detection of dysplasia and CIS, respectively, whereas the sensitivity of cystoscopy using HAL for these lesions remains in the range of 93% to 95% [8, 9]. The high recurrence rate requires frequent and prolonged surveillance by cystoscopy and makes bladder cancer the most expensive cancer to treat overall [10, 11]. According to the EAU Guidelines on NMIBC, the current treatment regime for low risk NMIBC patients is cystoscopy at 3 months after TUR, and if negative, then the next cystoscopy is advised done 9 months later and then yearly for 5 years [6]. For high risk NMIBC and CIS patients the standard surveillance is cystoscopy and urinary cytology at 3 months, and if negative, this is repeated every three months for 2 years, then half-yearly for up to 5 years, and then yearly [6]. In patients with an intermediate risk of progression, surveillance is in between that advised for the followup for low- and for high-risk patients [6]. Cytology is a noninvasive operator-dependent test often used in combination with cystoscopy, owing to a very high specificity of 96% (94–98; 95% CI), but with a low average sensitivity of 44% (38–51, 95% CI). Cytology has higher sensitivity for high-stage and high-grade tumors than for low-stage and low-grade tumors [12]. 

The perfect urinary marker of bladder cancer should reliably detect all tumors for the well-being of the patients and at the same time have a very high specificity to minimize false positive results. This is especially true when considering screening in general, but also when considering screenings of high risk groups and patients under suspicion of bladder cancer. Six markers are approved by the U.S. Food and Drug Administration (FDA) for diagnosis of bladder cancer in patients suspected of having bladder cancer, or for surveillance of bladder cancer. While they are all more sensitive than cytology, they are unable to replace cystoscopy. This paper will list the performance of cytology, the FDA-approved biomarkers NMP22, ImmunoCyt, and UroVysion, and investigational methylation urinary markers for the diagnosis and surveillance of bladder cancer. The performance of methylation markers will be compared with cytology and the FDA-approved markers. Furthermore, this paper will discuss whether the urinary methylation markers may replace cystoscopy, replace cytology as an adjunct to cystoscopy, or postpone the time between cystoscopies in the surveillance of NMIBC. Studies included in the review have been chosen based on the number of participants included, and the ability to determine whether primary lesions or recurrent lesions have been studied in the papers.

## 2. Cytology

Urinary cytology is the gold standard for noninvasive urinary diagnosis of bladder cancer, and the cytological examination is performed by a pathologist or cytologist who identifies cancer cells in the urine. The cells in the urine are classified into one of four categories: normal, atypical/indeterminate, suspicious, or malignant [13]. Urinary infections or other inflammatory conditions of the bladder may produce false positive results, and in addition the inter- and intraobserver variability of cytology are both high [14, 15]. Cytology has an overall test sensitivity of 44% (38–51, 95% CI) (Table 1), but low-grade tumors (grades I and II) have a lower test sensitivity than high-grade tumors. The test sensitivity of low-grade tumors is 27%, with a range from 0% to 93%, whereas high-grade tumors have a test sensitivity of 69%, with a range from 0% to 100%. Pooled estimates from 36 studies including 14,260 patients have established that the specificity of cytology is very high 96% (94–98; 95% CI) with a low false-positive interpretation [12]. In patients with a history of NMIBC the sensitivity is 38% (12–47; range) and the specificity is 94% (83–97; range) (Table 2) [12].

## 3. Commercially Available Bladder Cancer ****Markers

### 3.1. Nuclear Matrix Protein 22

The nuclear matrix protein 22 (NMP22) is a nuclear matrix protein that is found in all cells. During mitosis, the NMP 22 protein is found in the mitotic spindle, where it has an important role in the distribution of chromatin to daughter cells [16, 17]. The level of NMP22 in bladder cancer cell lines has been shown to be 25-fold more concentrated than in the healthy urothelium from a normal bladder [18]. In patients with bladder tumors, the NMP22 level was reported as 5 times greater than in individuals with no bladder malignancies [19]. False positive tests have been observed in individuals with benign inflammatory conditions, and several other nonmalignant conditions [20]. NMP22 was initially developed as a quantitative assay (NMP22 test), but was later transformed into the NMP22 BladderChek kit. The sensitivity and specificity of the NMP22 tests, counting 10,119 patients in 28 studies, are 68% (62–74; 95% CI) and 94% (83–97; 95% CI), respectively (Table 1). The NMP22 test has a higher sensitivity for high-grade tumors than for low-grade tumors. The test sensitivity for low-grade tumors is 50%, with a range from 0% to 86%, whereas the test sensitivity is 83%, with a range from 0% to 100%, for high-grade tumors [12]. In patients with a history of NMIBC, the test sensitivity is 69% (50–85; range) and the specificity is 81% (46–93; range) (Table 2) [12].

### 3.2. ImmunoCyt

ImmunoCyt combines cytology with an immunocytofluorescence technique based on a cocktail of monoclonal antibodies labeled with fluorescent markers. The monoclonal antibodies recognize a high molecular weight form of the carcinoembryonic antigen and two bladder tumor cell-associated mucins. The analysis is performed in a laboratory and requires a large number of exfoliated cells [21]. Applying a cut-off point of at least one green or one red fluorescent cell, the test sensitivity and specificity of the ImmunoCyt assay with 2,896 participating patients in eight studies are 84% (77–91; 95% CI) and 75% (68–83; 95% CI), respectively (Table 1). The test sensitivity for low-risk tumors is 81%, with a range from 55% to 90%, whereas high-risk tumors have a test sensitivity of 90%, with a range from 67% to 100% [12]. In patients with a history of NMIBC, the test sensitivity is 81% and the specificity is 75% (Table 2) [12].

### 3.3. UroVysion—Fluorescence In Situ Hybridization (FISH)

UroVysion is a multitarget fluorescence in situ hybridization (FISH) assay that utilizes DNA probes to identify aneuploidy in chromosomes 3, 7, 17, and loss of the 9p21 locus of the P16 tumor suppressor gene. The assay requires exfoliated cells in the urine. The probe binds to the complementary DNA in these cells, thereby visualizing the location of the targeted chromosomes. The recommended cutoff for a positive result for bladder cancer is usually defined as finding more than five urothelial cells with a gain of more than two chromosomes or ten cells with a gain of a single chromosome, or 12 or more cells with homozygous loss of the 9p21 locus. However, the cutoff may vary between institutions performing this assay. Applying this cutoff or a cutoff very close to this, the sensitivity and specificity of the ImmunoCyt assay with 2535 participating patients in 12 studies are 76% (65–84; 95% CI) and 85% (78–92; 95% CI), respectively (Table 1). The test sensitivity of low-risk tumors is 65%, with a range from 32% to 100%, whereas high-risk tumors have a test sensitivity of 95%, with a range from 50% to 100% [12]. In patients with a history of NMIBC, the test sensitivity is 64% and the specificity is 73% (Table 2) [12].

## 4. Investigational Bladder Cancer Markers 

Presently, there are several investigational urinary biomarkers of bladder cancer which are not yet commercially available, but the most promising will perhaps be approved as urinary markers in the future. A noteworthy group of these promising emerging markers is DNA methylation markers. These markers have some advantages that make them promising as tumor markers: (i) the DNA is quite stable; (ii) methylation can be detected by sensitive real-time PCR assays in a high through-put manner; (iii) results are not dependent on subjective analysis. The remainder of this paper will focus on DNA methylation markers and their application for diagnosis and surveillance in bladder cancer. 

### 4.1. DNA Methylation in Cancer

Cancer initiation and progression are driven by the accumulation of inherited or acquired DNA alterations. These changes may be genetic or epigenetic in nature [22]. Epigenetic changes are defined as heritable changes in gene function which do not involve changes in DNA sequence. Although DNA methylation and histone modifications (methylation, phosphorylation, acetylation) are all epigenetic modifications, only DNA methylation is included in the review. DNA methylation is, almost exclusively, the attachment of a methyl group to the 5th carbon in cytosine positioned just upstream of a guanosine (CpG). Most CpG sites are sparsely distributed throughout the genome and methylated, with the exception of CpG sites located in clusters, termed CpG islands. The majority of CpG islands are unmethylated and located within the promoter regions and exon 1 of more than 50% of all known genes [23–25]. CpG dinucleotides outside CpG islands are generally methylated in normal cells and undergo a substantial loss of DNA methylation in cancers. Increased genetic instability was observed in DNMT1 and DNMT3b double knock-out cells [26] and cells with hypomorphic allele of DNA methyltransferase 1 [27]. Mechanisms linked to hypomethylation induced genetic instability include decondensation of chromatin into recombination permissive conformation [28] and activation of retrotransposon elements [29]. Furthermore, hypomethylation of enhancer sites or other regulatory sites repressed by methylation may trigger increased expression of cancer promoting genes [22, 30, 31]. CpG sites within CpG islands are usually in an unmethylated state, permissive to transcription in normal cells, but become hypermethylated at certain promoters in cancers. DNA methylation within CpG islands, located in promoter regions, is involved in the silencing of DNA transcription together with histone modifications [32]. Development, genomic imprinting, and X-chromosome inactivation are critical normal processes in which DNA methylation occurs [33–35]. Alterations in epigenetic control have been associated with several human pathologic conditions including cancer [36]. 

### 4.2. DNA Methylation and Bladder Cancer: Tumor Tissue

With the use of microarray technology, the number of genes reported to be aberrantly methylated in bladder cancer tissue is now in the thousands [37, 38]. It has been shown that high-risk tumors, generally, have more hypermethylated genes than do low-risk tumors [37, 38]. Wolff and colleagues suggest two different epigenetic pathways depending on whether the tumor is muscle-invasive or not [37]. An important finding for the use of methylation as a tumor marker comes from a study with 10 patients. A study of metachronous tumors from these 10 patients indicated that the change in tumor methylation from normal urothelium was stable within patients [38]. Multiple studies by separate groups present data that aberrant methylation is associated with the stage and grade of the tumors, as well as the recurrence rate and risk of progression [38–48]. Transcriptional inactivation by CpG island promoter hypermethylation is a well-established mechanism for gene silencing in cancer including bladder cancer [46, 49–58]. Many genes have been reported aberrantly methylated in bladder cancer, but relatively few of the genes have been characterized with respect to the function of the attendant gene silencing. 

Age-dependent methylation of CpG islands occurs in a tissue-specific manner in normal appearing tissue [59, 60], and in bladder cancer increased methylation of CpG islands has been reported to be associated with high age of the patients [44, 61]. Examples of CpG islands in which methylation is associated with high age include CCNA1, PGP9, and CCND2 [44]. These findings make it of paramount importance to include age-matched controls when identifying diagnostic DNA methylation markers for bladder cancer. 

## 5. DNA Methylation and Bladder Cancer: Urine 

### 5.1. Diagnosis of Urothelial Carcinoma of the Bladder

Several studies have reported results indicating that methylation markers, applied to DNA from voided urine or bladder washes, can be used for the diagnosis of bladder cancer (Table 3). Nine studies presented results from patients with a suspicion of bladder cancer [57, 62–69], whereas other studies included a mixture of samples from patients with a suspicion of bladder cancer, and patients previously diagnosed with bladder cancer, or the information was not reported [21, 38, 70–77]. Patients with a confirmed suspicion of bladder cancer will generally have larger tumors that shed more cells into the urine than patients under surveillance for bladder cancer. Cancer cells shed from tumors within the bladder may be of low or high grade. Low-grade tumors are less likely to shed many cells into the urine as their high-grade counterparts because the high-grade tumors have weaker intercellular attachments [78]. Depending on the detection method this may influence the sensitivity of the urinary markers.

The large variation of cell types in the urine necessitates that the DNA methylation markers are cancer specific and uninfluenced by hematuria, bladder infections, or other benign bladder conditions. Many of the studies have included individuals with a mixture of benign urothelial conditions to insure the cancer specificity of the methylation markers, although some studies have either not done this or were not reported.

The first study demonstrating the feasibility of diagnosing bladder cancer through methylation using DNA from voided urine came from Chan and colleagues in 2002 [71]. They analyzed urine sediments from voided urine from 22 patients and 17 age- and sex-matched controls by methylation sensitive PCR (MSP). The analysis was based on a panel of markers (DAPK, RAR*β*, E-cadherin, and p16) that achieved a sensitivity of 91% and a specificity of 76% (Table 3) [71]. In the same study, the sensitivity and specificity of cytology were 46% and 100%, respectively. Subsequently, many studies have reported methylation markers with an increased sensitivity, but a lower specificity than cytology. Investigations of matched tumor and urine samples showed no false positive urine samples when the tumor was negative for methylation, indicating the specificity of the markers. Unfortunately, Chan et al. did not provide any information about bladder conditions within the control group. 

The fact that DNA methylation markers may be as specific as cytology was revealed in a study by Dulaimi et al. with 45 bladder cancer patients and 21 healthy age-matched individuals including 9 individuals with cystitis. The sensitivity was only 87%, but the specificity was 100% (Table 3). The concordance between methylation in the tumor and the matched urine was very high, and no urine sample was positive without simultaneous tumor methylation [73].

Quantitative real-time PCR was introduced by Friedrich et al., who investigated 37 bladder cancer patients undergoing radical cystectomy, 10 age-matched cancer-free individuals, and 10 non-age-matched cancer-free individuals. A panel of markers (DAPK, BCL2, and TERT) achieved a sensitivity of 78% and a specificity of 100% (Table 3) [74]. Currently, real-time PCR is the most applied technique, owing to the low DNA requirement and high sensitivity, compared with MSP.

A different technique was applied by Reinert and coworkers when they used methylation-sensitive high-resolution melting (MS-HRM) PCR to study voided urine from 119 bladder cancer patients and 59 individuals with no history of bladder cancer. The controls were from patients with benign prostatic hyperplasia (BPH) or bladder stones, and 19 patients were suspected of having a bacterial infection. In that study, a four-marker panel (ZNF154, HOXA9, POU4F2, and EOMES) achieved a sensitivity of 84% and a specificity of 96% (Table 3) [38]. Recently, a validation of ZNF154, HOXA9, POU4F2, and EOMES by real-time PCR (MethyLight) using DNA from 184 bladder cancer patients and 35 control individuals resulted in an increased sensitivity of the individual markers by eight to twenty-five percentage points (Table 2) [38, 65]. The increased test sensitivity is probably due to more sensitive MethyLight primers, as the patient cohorts are comparable. 

Notably, a prospective multicenter study with both training and validation sets with a total of 83 bladder cancer patients and 178 individuals with no history of bladder cancer achieved a sensitivity of 90% and a specificity of 93%. The control patients were all diagnosed with benign urologic diseases (Table 2) [66].

Several additional studies have confirmed that urinary markers may be highly sensitive for the detection of bladder cancer and at the same time very specific, even when examining patients with other urological diseases (Table 3). Lately, numerous methylation markers identified by screening of bladder cancer tissue samples or cell lines show high sensitivity and specificity when translated to BC detection in urine [38, 64, 66, 72, 77]. Bladder marker studies require age-matched controls to ensure that the observed methylation is tumor specific, since methylation within CpG islands may increase with age in a tissue-specific manner [60]. Most studies have included age-matched controls, but in the study by Lin et al. the median age between bladder cancer patients and healthy individuals varied by more than 30 years [76]. Many studies have reported a high concordance between tumor tissue and urine specimen methylation, indicating that the methylation detected was specific for bladder cancer cells and did not reflect methylation of DNA from other sources in the urinary system [21, 38, 67, 70–73, 75–77, 81]. Deviating from this was Vinci et al. who found the methylation status of DAPK to be discordant between tumor and urine sediments [21]. Several methylation markers have shown higher sensitivity with increasing stage [21, 38, 68, 75, 81] and grade [21, 38, 65, 70, 81]. Two studies have reported that the methylation markers reported in their studies were not independent of each other [38, 75]. This indicates that one single methylation mechanism may account for the majority of the methylation alterations analysed in those studies.

Methylation markers for bladder cancer diagnosis are still at an early stage compared with the FDA approved markers. Most of the reported markers have been tested on cohorts that varied greatly between studies. In addition to this, many markers are lacking validation in independent prognostic experiments with predetermined cut-off values. Independent validation experiments will often achieve lower test sensitivity and/or lower test specificity, as the cut-off values from the initial experiment were fitted to the data. With that said, comparing the methylation markers with cytology, it is evident that the methylation markers are more sensitive than cytology, and for the some of the markers the specificity is even comparable with cytology (Table 2). Nevertheless, methylation markers have not entered the clinical setting as diagnostic markers of bladder cancer instead of cystoscopy or as a supplement to cystoscopy or cytology. 

### 5.2. Urinary DNA Methylation Markers for Surveillance of Bladder Cancer

Patients previously diagnosed with bladder cancer are included in a treatment regimen with frequent follow-up cystoscopy examinations, and perhaps cytology as an adjunct. Cystoscopy is an invasive procedure that is costly for the healthcare system and unpleasant for the patient. The search for a noninvasive DNA methylation marker for the diagnosis of bladder cancer was initiated more than a decade ago by Chan and coworkers [71], whereas the search for a diagnostic DNA methylation marker for surveillance of bladder cancer patients only started in 2008 with a study by Rouprêt et al. [79]. They designed a control group from bladder cancer patients with a negative cystoscopy and/or biopsies. In this small study with 40 patients of which 15 experienced a recurrence, they reported the sensitivity of an 11 gene panel to be 86%, while the specificity was as low as 8% (Table 2). In a more recent study by Zuiverloon et al. they developed a four gene markers panel by screening NMIBC tissue, urine from nonbladder cancer patients, and blood from bladder cancer patients for methylation of 37 genes. In the validation cohort with 94 bladder cancer patients under surveillance, the sensitivity of this panel of urinary markers was 72% and the specificity was 55% (Table 2) [80]. In a most recent and yet unpublished study by Reinert et al. [65], they tested six methylation markers that have previously been shown to be able to differentiate between bladder cancer patients and individuals with no history of bladder cancer with high sensitivity and specificity [38]. When including only urine samples from patients that had previously tested positive for methylation, and analyzing 206 voided urine samples from patients under surveillance for bladder cancer, they achieved a test sensitivity of 93% and a specificity of 47% of a single marker when comparing to cystoscopy carried out at the same visit to the clinic. Including cystoscopy results during a 12-month follow-up period in the analysis, they found many of the samples formerly classified as false positives to be true positives (anticipatory positives). The adjusted methylation marker sensitivity and specificity were 94% and 66%, respectively. Importantly, in these settings all six markers were independent of stage and grade.

Anticipatory positives are results that show a positive finding preceding visual evidence of a bladder tumor. The term “anticipatory positive” is debatable, due to the high recurrence rate in bladder cancer. However, a prognostic study with 250 recurrent cases by Yoder and colleagues focusing on anticipatory findings showed that of 148 patients with negative cystoscopy, 5% of UroVysion negative and 65% of UroVysion positive results experienced a recurrent urinary cancer within 29 months after cystoscopy [82]. In a similar manner, the anticipatory effect has been shown for DNA methylation markers by Reinert and colleagues, who found that 4% of methylation-negative and 63% of methylation-positive patients relapse within 12 months [65]. 

### 5.3. Urinary DNA Methylation Markers for Prediction of Recurrence Free Survival

While several studies have focused on diagnostic markers and markers of surveillance, the potential of urinary methylation markers as prognostic markers of recurrence has just recently been suggested in a single and yet unpublished study by Reinert et al. [65]. They reported that detection of DNA methylation of ZNF154, HOXA9, POU4F2, TWIST1, or VIM was significantly associated with future recurrences, with hazard ratios ranging from 7.8 to 13.9. They speculated that the prognostic value of the methylation markers was connected with the existence of a DNA hypermethylation field disease as reported previously [37]. 

## 6. Conclusion

Early diagnosis of bladder cancer, and careful followup for detection of recurrences after initial treatment, are main tasks of current urological research. The high rate of recurrences and the prolonged followup by cystoscopy and cytology make bladder cancer the most expensive cancer to treat, overall. However, by utilizing noninvasive urinary markers, it may be possible to improve the diagnosis of new cancers as well as improve the management of NMIBC. NMP22, UroVysion, and ImmunoCyt are well-examined urinary markers. NMP22 and UroVysion are both FDA-approved tests for initial diagnosis of bladder cancer in patients with a suspicion of bladder cancer and for surveillance of bladder cancer; however, ImmunoCyt is only approved for the surveillance of bladder cancer in conjunction with urinary cytology and cystoscopy. All three markers have a higher sensitivity than cytology, but a lower specificity (Table 1). Furthermore, the specificity of NMP22 and ImmunoCyt are influenced by urinary conditions other than bladder cancer, which makes them unusable in many situations (Table 1). 

The methylation markers are not as well studied as the FDA-approved markers, and none have been approved by the FDA for the diagnosis or surveillance of bladder cancer. Weaknesses of the methylation studies include (1) many small studies with a limited number of participants; (2) different methods for analyzing methylation between studies making comparison of markers difficult; (3) low homogeneity of study population regarding stage and grade of the patients; (4) lack of information about adjuvant intravesical therapy; (5) heterogeneous patients populations, as many studies include patients under suspicion for bladder cancer as well as patients with a history of bladder cancer; (6) absence of age- and sex-matched reference groups; (7) retrospectively collected samples that may be biased regarding availability of material for analysis. With these reservations in mind, most methylation markers show higher sensitivity than cytology, but often at the cost of a lower specificity (Table 2). For diagnosis of bladder cancer in patients under suspicion of bladder cancer, the most sensitive and specific DNA methylation markers are probably preferable compared with the FDA-approved markers, owing to the higher sensitivity and specificity of the methylation marker, in addition to the fact that most of the DNA methylation markers have been shown to be uninfluenced by other benign bladder conditions or benign prostatic hyperplasia. 

**Figure 1:**
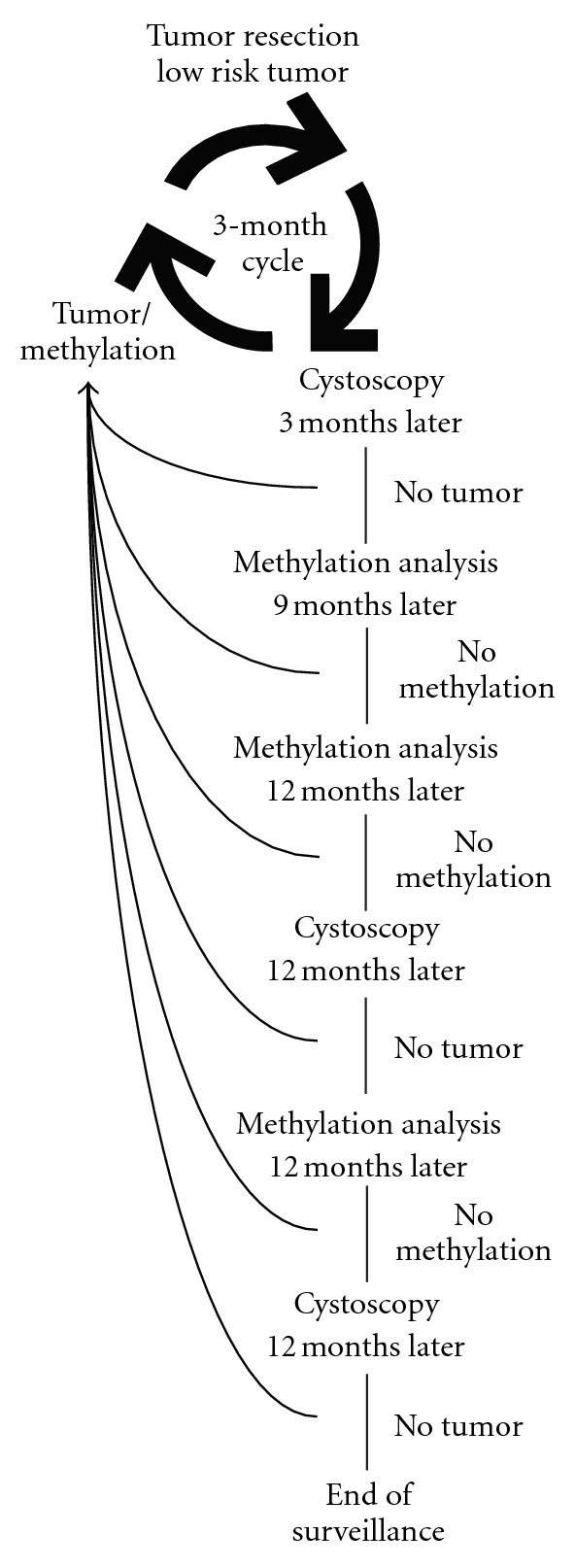
Follow-up model for low- and medium-risk NMIBC in which methylation markers were applied to prolong time between cystoscopies.

For surveillance of bladder cancer patients, DNA methylation markers have the highest sensitivity (94%) followed by ImmunoCyt (81%), NMP22 (69%), UroVysion (64%), and cytology (38%). Cytology has the highest specificity (94%) followed by NMP22 (81%), ImmunoCyt (75%), UroVysion (73%), and DNA methylation markers (66%). In situations where high test sensitivity is important, DNA methylation markers ought to be the test of choice, whereas if a high specificity is important, cytology should be the preferred marker. Considering the question whether or not a urinary marker may postpone the time between cystoscopies or replace cytology in surveillance of NMIBC, the answer must be that some of the methylation markers are more than capable, because the DNA methylation markers have higher sensitivity than white light cystoscopy and cytology. This assumes that the current results can be validated in independent prognostic studies. In addition, it will be important to have some comparison studies in which the DNA methylation markers are compared with established markers using standardized assays and cut-off values. In a study by Yossepowitch et al. they conclude that 75% of patients under surveillance for bladder cancer would accept the result of a urine marker test as a replacement for cystoscopy, if the sensitivity of the marker was at least 95% [83]. It is worth considering that the patients interviewed were expecting the sensitivity of cystoscopy to be 100%, which is not true in all cases. A suggested surveillance regime for low- and medium-risk NMIBC could be to replace the cystoscopies at 12, 24, and 48 months, after the removal of the tumor, with methylation markers (Figure 1). The potential of the methylation markers is unmatched by cytology and the FDA-approved markers with respect to detecting recurrent tumors. However, until additional prospective studies of suitable proportions have validated the promising results, surveillance will continue to consist of cystoscopy and cytology. 

## 7. Future Perspectives

Detecting bladder cancer using diagnostic or surveillance markers remains a challenge, as none of the currently approved markers can replace cystoscopy or prolong the time between cystoscopies. The field of methylation biomarkers is promising, but requires more prospective multicenter studies that explicitly clarify the purpose of the study as screening, diagnosis of primary disease, or surveillance. In addition, these studies should include the appropriate patients in order to validate the current findings in an unbiased manner. Currently, at least one such study combining methylation markers and FGFR3 mutational markers for surveillance of bladder cancer is ongoing (UROMOL, FP7 EU study). This study investigates urine samples from more than 1200 patients with bladder cancer in several clinical centers in Europe. 

Recent technological advances within the field of methylation analysis, like the Illumina Infinium HumanMethylation450 BeadChip, or some of the many next generation sequencing-based methylation analyses, for example, whole-genome shotgun bisulfite sequencing, may uncover even more sensitive and specific markers in the future. Nevertheless, the emphasis should be to develop existing markers and in parallel identify new promising methylation markers in order to increase survival and the quality of life for bladder cancer patients.

## Figures and Tables

**Table 1:** Sensitivity and specificity of cytology, NMP22, ImmunoCyt, and UroVysion when no distinguishment is made between patients with suspicion of bladder cancer and patients previously diagnosed with bladder cancer.

Test	Samples/studies	Sensitivity, % (95% CI)	Specificity, % (95% CI)	Interference by other bladder conditions	Comments	Reference
Cytology^a^	14260/36	44 (38–51)	96 (94–98)	Yes	Subjective judgement	[12]
NMP22^b^	10119/28	68 (62–74)	79 (74–84)	Yes	Cutoff was ≥10 U/mL for positive test result	[12]
ImmunoCyt	2896/8	84 (77–91)	75 (68–83)	Yes	At least one green or one red fluorescent cell	[12]
UroVysion	2535/12	76 (65–84)	85 (78–92)	No	Cutoffs may vary between studies	[12]

^a^ Voided urine only.

^b^ Including five studies using NMP22 BladderChek.

**Table 2:** Performance of cytology and biomarkers in studies that include patients with suspicion of bladder cancer or patients previously diagnosed with bladder cancer.

Test/marker	Suspicion^a^/previous^b^ history of BC	Samples/ studies	Sensitivity, % Median (range)	Specificity, % Median (range)	Reference
Cytology	Suspicion	3331/7	44% (16–100)	96 (94–98)	[12]
	Previous history of BC	4495/6	38% (12–47)	94 (83–97)	

NMP22	Suspicion	1893/4	71 (56–100)	86 (80–87)	[12]
	Previous history of BC	4284/7	69 (50–85)	81 (46–93)	

ImmunoCyt	Suspicion	280/1	85	88	[12]
	Previous history of BC	326/1	81	75	

UroVysion	Suspicion	497/1	69	78	[12]
	Previous history of BC	250/1	64	73	

PMF1	Suspicion	118/1	65	95	[58, 62]
	Previous history of BC				

Myopodin	Suspicion	164/1	65	80	[57]
	Previous history of BC				

IRF8, p14, and sFRP1	Suspicion	45/1	85	95	[63]
	Previous history of BC	4/1	100	—^c^	

MYO3A, CA10, NKX6-2, DBC1, and SOX11 or PENK	Suspicion	198/1	85	95	[64]
	Previous history of BC	40/1	85	—	

ZNF154, EOMES, HOXA9, POU4F2, TWIST1, and VIM	Suspicion	79/1	98	100	[38, 65]
	Previous history of BC	206/1	94	66	

TWIST1 and NID2	Suspicion	278/1	90	93	[66]
	Previous history of BC				

RASSF1A, E-cadherin,	Suspicion				[79]
APC, DAPK, MGMT, BCL2, h-TERT, EDNRB, WIF1, TNFRSF25, and IGFBP3	Previous history of BC	40/1	86	8	

APC, RASFF1A, RARB, DBC1, SFRP1, SFRP2, SFRP4, SFRP5	Suspicion	146/1	52	100	[67]
	Previous history of BC				

SFRP1, SFRP2, SFRP4, SFRP5, VIF-1, and DKK3	Suspicion	264/1	61.1	93.3	[50, 68]
	Previous history of BC				

RASSF1a, E-cad, and APC	Suspicion	104/1	69	60	[39, 69]
	Previous history of BC				

APC_a, TERT_a, TERT_b, and EDNRB	Suspicion				[80]
	Previous history of BC	94	72	55	

^a^Only studies that include patients with a suspicion of bladder cancer.

^b^Only studies that include patients with a previous history of bladder cancer.

^c^Specificity in studies using healthy individuals as controls is not shown.

**Table 3:** Performance of DNA methylation markers in studies that include patients with suspicion of bladder cancer and patients previously diagnosed with bladder cancer.

Test	Method	Patients^a^/ studies	Sensitivity, %	Specificity, %	Interference by other bladder conditions	Reference
PMF1	MSP	118/1	65	95	No	[58, 62]
Myopodin	MSP	164/1	65	80	No	[57]
RASSF1A	MSP	24/1	50	100	Not done	[70]
DAPK, RAR*β*, E-cadherin, and p16	MSP	39/1	91	76	Not done	[71]
IRF8, p14, and sFRP1	qMSP	49/1	87	95	No information	[63]
MYO3A, CA10, NKX6-2, DBC1, and SOX11 or PENK	qMSP	238/1	85	95	No	[64]
GDF15, TMEFF2, and VIM	qMSP	110/1	94	90	Not done	[72]
APC, RASSF1A, and p14^ARF^	MSP	66/1	87	100	No	[73]
DAPK, BCL2, and TERT	qMSP	57/1	78	100	Not done	[42, 74]
CDKN2A, ARF, MGMT, and GSTP1	qMSP	269/1	69	100	No	[41, 75]
RASSF1A, p14, and E-cadherin	MSP	66/1	80	100	Not done	[76]
ZNF154, HOXA9, POU4F2, and EOMES	MS-HRM	174/1	84	96	No	[38, 65]
TWIST1 and NID2	qMSP	278/1	90	93	No	[66]
APC, RASFF1A, RARB, DBC1, SFRP1, SFRP2, SFRP4, SFRP5	qMSP	146/1	52	100	No	[67]
SFRP1, SFRP2, SFRP4, SFRP5, VIF-1, and DKK3	MSP	264/1	61.1	93.3	Not done	[50, 68]
BCL2 and hTERT	qMSP	213/1	76	98	No	[21]
RASSF1a, E-cad, and APC	qMSP	104/1	69	60	No	[39, 69]
SALL3, CFTR, ABCC6, HPR1, RASSF1A, MT1A, RUNX3, ITGA4, BCL2, ALX4, MYOD1, DRM, CDH13, BMP3B, CCNA1, RPRM, MINT1, and BRCA1	MSP	159/1	92	88	No	[77]

^a^Including both patients with BC and individuals with no history of bladder cancer.

^b^Not applicable.

## References

[1] Jemal A, Siegel R, Xu J, Ward E (2010). Cancer statistics, 2010. *CA Cancer Journal for Clinicians*.

[2] Silverman DT, Levin LI, Hoover RN, Hartge P (1989). Occupational risks of bladder cancer in the United States: I. White men. *Journal of the National Cancer Institute*.

[3] Clavel J, Cordier S, Boccon-Gibod L, Hemon D (1989). Tobacco and bladder cancer in males: increased risk for inhalers and smokers of black tobacco. *International Journal of Cancer*.

[4] Kiemeney LA, Thorlacius S, Sulem P (2008). Sequence variant on 8q24 confers susceptibility to urinary bladder cancer. *Nature Genetics*.

[5] Altekruse SF, Krapcho M, Neyman N (2010). *SEER Cancer Statistics Review, 1975–2007*.

[6] Babjuk M, Oosterlinck W, Sylvester R (2011). EAU guidelines on non-muscle-invasive urothelial carcinoma of the bladder, the 2011 update. *European Urology*.

[7] Sylvester RJ, Van Der Meijden APM, Oosterlinck W (2006). Predicting recurrence and progression in individual patients with stage Ta T1 bladder cancer using EORTC risk tables: A combined analysis of 2596 patients from seven EORTC trials. *European Urology*.

[8] Grossman HB, Gomella L, Fradet Y (2007). A Phase III, multicenter comparison of hexaminolevulinate fluorescence cystoscopy and white light cystoscopy for the detection of superficial papillary lesions in patients with bladder cancer. *Journal of Urology*.

[9] Jocham D, Witjes F, Wagner S (2005). Improved detection and treatment of bladder cancer using hexaminolevulinate imaging: a prospective, phase III multicenter study. *Journal of Urology*.

[10] Avritscher EBC, Cooksley CD, Grossman HB (2006). Clinical model of lifetime cost of treating bladder cancer and associated complications. *Urology*.

[11] Botteman MF, Pashos CL, Redaelli A, Laskin B, Hauser R (2003). The health economics of bladder cancer: a comprehensive review of the published literature. *PharmacoEconomics*.

[12] Mowatt G, Zhu S, Kilonzo M (2010). Systematic review of the clinical effectiveness and cost-effectiveness of photodynamic diagnosis and urine biomarkers (FISH, ImmunoCyt, NMP22) and cytology for the detection and follow-up of bladder cancer. *Health Technology Assessment*.

[13] Murphy WM (1990). Current status of urinary cytology in the evaluation of bladder neoplasms. *Human Pathology*.

[14] Talwar R, Sinha T, Karan SC (2007). Voided urinary cytology in bladder cancer: is it time to review the indications?. *Urology*.

[15] Tritschler S, Sommer ML, Straub J (2010). Urinary cytology in Era of fluorescence endoscopy: redefining the role of an established method with a new reference standard. *Urology*.

[16] Berezney R, Coffey DS (1974). Identification of a nuclear protein matrix. *Biochemical and Biophysical Research Communications*.

[17] Fey EG, Bangs P, Sparks C, Odgren P (1991). The nuclear matrix: defining structural and functional roles. *Critical Reviews in Eukaryotic Gene Expression*.

[18] Carpinito GA, Stadler WM, Briggman JV (1996). Urinary nuclear matrix protein as a marker for transitional cell carcinoma of the urinary tract. *Journal of Urology*.

[19] Jamshidian H, Kor K, Djalali M (2008). Urine concentration of nuclear matrix protein 22 for diagnosis of transitional cell carcinoma of bladder. *Urology Journal*.

[20] Ponsky LE, Sharma S, Pandrangi L (2001). Screening and monitoring for bladder cancer: refining the use of NMP22. *Journal of Urology*.

[21] Vinci S, Giannarini G, Selli C (2011). Quantitative methylation analysis of BCL2, hTERT, and DAPK promoters in urine sediment for the detection of non-muscle-invasive urothelial carcinoma of the bladder: a prospective, two-center validation study. *Urologic Oncology*.

[22] Esteller M (2008). Molecular origins of cancer: epigenetics in cancer. *New England Journal of Medicine*.

[23] Takai D, Jones PA (2002). Comprehensive analysis of CpG islands in human chromosomes 21 and 22. *Proceedings of the National Academy of Sciences of the United States of America*.

[24] Jones PA, Baylin SB (2002). The fundamental role of epigenetic events in cancer. *Nature Reviews Genetics*.

[25] Krakowski M, Abdelmalik R, Mocnik L, Krahl T, Sarvetnick N (2002). Cancer as an epigenetic disease: DNA methylation and chromatin alterations in human tumours. *Journal of Pathology*.

[26] Karpf AR, Matsui SI (2005). Genetic disruption of cytosine DNA methyltransferase enzymes induces chromosomal instability in human cancer cells. *Cancer Research*.

[27] Eden A, Gaudet F, Waghmare A, Jaenisch R (2003). Chromosomal instability and tumors promoted by DNA hypomethylation. *Science*.

[28] Tuck-Muller CM, Narayan A, Tsien F (2000). DNA hypomethylation and unusual chromosome instability in cell lines from ICF syndrome patients. *Cytogenetics and Cell Genetics*.

[29] Steinhoff C, Schulz WA (2003). Transcriptional regulation of the human LINE-1 retrotransposon L1.2B. *Molecular Genetics and Genomics*.

[30] Jones PA, Gonzalgo ML (1997). Altered DNA methylation and genome instability: a new pathway to cancer?. *Proceedings of the National Academy of Sciences of the United States of America*.

[31] Ehrlich M (2002). DNA methylation in cancer: too much, but also too little. *Oncogene*.

[32] Jones PA, Baylin SB (2007). The epigenomics of cancer. *Cell*.

[33] Migeon BR (1992). Concerning the role of X-inactivation and DNA methylation in fragile X syndrome. *American Journal of Medical Genetics*.

[34] Li E, Bestor TH, Jaenisch R (1992). Targeted mutation of the DNA methyltransferase gene results in embryonic lethality. *Cell*.

[35] Li E, Beard C, Jaenisch R (1993). Role for DNA methylation in genomic imprinting. *Nature*.

[36] Egger G, Liang G, Aparicio A, Jones PA (2004). Epigenetics in human disease and prospects for epigenetic therapy. *Nature*.

[37] Wolff EM, Chihara Y, Pan F (2010). Unique DNA methylation patterns distinguish noninvasive and invasive urothelial cancers and establish an epigenetic field defect in premalignant tissue. *Cancer Research*.

[38] Reinert T, Modin C, Castano FM (2011). Comprehensive genome methylation analysis in bladder cancer: identification and validation of novel methylated genes and application of these as urinary tumor markers. *Clinical Cancer Research*.

[39] Yates DR, Rehman I, Abbod MF (2007). Promoter hypermethylation identifies progression risk in bladder cancer. *Clinical Cancer Research*.

[40] Jarmalaite S, Jankevicius F, Kurgonaite K, Suziedelis K, Mutanen P, Husgafvel-Pursiainen K (2008). Promoter hypermethylation in tumour suppressor genes shows association with stage, grade and invasiveness of bladder cancer. *Oncology*.

[41] Hoque MO, Begum S, Brait M (2008). Tissue inhibitor of metalloproteinases-3 promoter methylation is an independent prognostic factor for bladder cancer. *Journal of Urology*.

[42] Friedrich MG, Chandrasoma S, Siegmund KD (2005). Prognostic relevance of methylation markers in patients with non-muscle invasive bladder carcinoma. *European Journal of Cancer*.

[43] Catto JWF, Azzouzi AR, Rehman I (2005). Promoter hypermethylation is associated with tumor location, stage, and subsequent progression in transitional cell carcinoma. *Journal of Clinical Oncology*.

[44] Brait M, Begum S, Carvalho AL (2008). Aberrant promoter methylation of multiple genes during pathogenesis of bladder cancer. *Cancer Epidemiology Biomarkers and Prevention*.

[45] Tada Y, Wada M, Taguchi KI (2002). The association of Death-associated Protein Kinase hypermethylation with early recurrence in superficial bladder cancers. *Cancer Research*.

[46] Kim WJ, Kim EJ, Jeong P (2005). RUNX3 inactivation by point mutations and aberrant DNA methylation in bladder tumors. *Cancer Research*.

[47] Christoph F, Weikert S, Kempkensteffen C (2006). Regularly methylated novel pro-apoptotic genes associated with recurrence in transitional cell carcinoma of the bladder. *International Journal of Cancer*.

[48] Kandimalla R, Van Tilborg AAG, Kompier LC (2012). Genome-wide analysis of CpG Island methylation in bladder cancer identified TBX2, TBX3, GATA2, and ZIC4 as pTa-specific prognostic markers. *European Urology*.

[49] Veerla S, Panagopoulos I, Jin Y, Lindgren D, Höglund M (2008). Promoter analysis of epigenetically controlled genes in bladder cancer. *Genes Chromosomes and Cancer*.

[50] Urakami S, Shiina H, Enokida H (2006). Epigenetic inactivation of Wnt inhibitory factor-1 plays an important role in bladder cancer through aberrant canonical Wnt/*β*-catenin signaling pathway. *Clinical Cancer Research*.

[51] Stoehr R, Wissmann C, Suzuki H (2004). Deletions of chromosome 8p and loss of sFRP1 expression are progression markers of papillary bladder cancer. *Laboratory Investigation*.

[52] Sobti RC, MalekZadeh K, Nikbakht M, Sadeghi IA, Shekari M, Singh SK (2010). Hypermethylation-mediated partial transcriptional silencing of DAP-kinase gene in bladder cancer. *Biomarkers*.

[53] Mori K, Enokida H, Kagara I (2009). CpG hypermethylation of collagen type I *α* 2 contributes to proliferation and migration activity of human bladder cancer. *International Journal of Oncology*.

[54] Lokeshwar VB, Gomez P, Kramer M (2008). Epigenetic regulation of HYAL-1 hyaluronidase expression: identification of HYAL-1 promoter. *Journal of Biological Chemistry*.

[55] Lee MG, Kim HY, Byun DS (2001). Frequent epigenetic inactivation of RASSF1A in human bladder carcinoma. *Cancer Research*.

[56] Khin SS, Kitazawa R, Win N (2009). BAMBI gene is epigenetically silenced in subset of high-grade bladder cancer. *International Journal of Cancer*.

[57] Cebrian V, Alvarez M, Aleman A (2008). Discovery of myopodin methylation in bladder cancer. *Journal of Pathology*.

[58] Aleman A, Adrien L, Lopez-Serra L (2008). Identification of DNA hypermethylation of SOX9 in association with bladder cancer progression using CpG microarrays. *British Journal of Cancer*.

[59] Issa J-P (1999). Aging, DNA methylation and cancer. *Critical Reviews in Oncology/Hematology*.

[60] Christensen BC, Houseman EA, Marsit CJ (2009). Aging and environmental exposures alter tissue-specific DNA methylation dependent upon CPG island context. *PLoS Genetics*.

[61] Marsit CJ, Houseman EA, Schned AR, Karagas MR, Kelsey KT (2007). Promoter hypermethylation is associated with current smoking, age, gender and survival in bladder cancer. *Carcinogenesis*.

[62] Aleman A, Cebrian V, Alvarez M (2008). Identification of PMF1 methylation in association with bladder cancer progression. *Clinical Cancer Research*.

[63] Chen PC, Tsai MH, Yip SK (2011). Distinct DNA methylation epigenotypes in bladder cancer from different Chinese sub-populations and its implication in cancer detection using voided urine. *BMC Medical Genomics*.

[64] Chung W, Bondaruk J, Jelinek J (2011). Detection of bladder cancer using novel DNA methylation biomarkers in urine sediments. *Cancer Epidemiology Biomarkers and Prevention*.

[65] Reinert T Diagnosis and prediction of bladder cancer recurrence based on urinary levels of ZNF154, EOMES, HOXA9, POU4F2, TWIST1, and VIM hypermethylation.

[66] Renard I, Joniau S, van Cleynenbreugel B (2010). Identification and validation of the methylated TWIST1 and NID2 genes through real-time methylation-specific polymerase chain reaction assays for the noninvasive detection of primary bladder cancer in urine samples. *European Urology*.

[67] Serizawa RR, Ralfkiær U, Steven K (2011). Integrated genetic and epigenetic analysis of bladder cancer reveals an additive diagnostic value of FGFR3 mutations and hypermethylation events. *International Journal of Cancer*.

[68] Urakami S, Shiina H, Enokida H (2006). Combination analysis of hypermethylated Wnt-antagonist family genes as a novel epigenetic biomarker panel for bladder cancer detection. *Clinical Cancer Research*.

[69] Yates DR, Rehman I, Meuth M, Cross SS, Hamdy FC, Catto JWF (2006). Methylational urinalysis: a prospective study of bladder cancer patients and age stratified benign controls. *Oncogene*.

[70] Chan MWY, Chan LW, Tang NLS (2003). Frequent hypermethylation of promoter region of RASSF1A in tumor tissues and voided urine of urinary bladder cancer patients. *International Journal of Cancer*.

[71] Chan MWY, Chan LW, Tang NLS (2002). Hypermethylation of multiple genes in tumor tissues and voided urine in urinary bladder cancer patients. *Clinical Cancer Research*.

[72] Costa VL, Henrique R, Danielsen SA (2010). Three epigenetic biomarkers, GDF15, TMEFF2, and VIM, accurately predict bladder cancer from DNA-based analyses of urine samples. *Clinical Cancer Research*.

[73] Dulaimi E, Uzzo RG, Greenberg RE, Al-Saleem T, Cairns P (2004). Detection of bladder cancer in urine by a tumor suppressor gene hypermethylation panel. *Clinical Cancer Research*.

[74] Friedrich MG, Weisenberger DJ, Cheng JC (2004). Detection of methylated apoptosis-associated genes in urine sediments of bladder cancer patients. *Clinical Cancer Research*.

[75] Hoque MO, Begum S, Topaloglu O (2006). Quantitation of promoter methylation of multiple genes in urine DNA and bladder cancer detection. *Journal of the National Cancer Institute*.

[76] Lin HH, Ke HL, Huang SP, Wu WJ, Chen YK, Chang LL (2010). Increase sensitivity in detecting superficial, low grade bladder cancer by combination analysis of hypermethylation of E-cadherin, p16, p14, RASSF1A genes in urine. *Urologic Oncology*.

[77] Yu J, Zhu T, Wang Z (2007). A novel set of DNA methylation markers in urine sediments for sensitive/specific detection of bladder cancer. *Clinical Cancer Research*.

[78] Villicana P, Whifting B, Goodison S, Rosser CJ (2009). Urine-based assays for the detection of bladder cancer. *Biomarkers in Medicine*.

[79] Rouprêt M, Hupertan V, Yates DR (2008). A comparison of the performance of microsatellite and methylation urine analysis for predicting the recurrence of urothelial cell carcinoma, and definition of a set of markers by Bayesian network analysis. *British Journal of Urology International*.

[80] Zuiverloon TC, Beukers W, Van Der Keur KA (2012). A methylation assay for the detection of non-muscle-invasive bladder cancer (NMIBC) recurrences in voided urine. *British Journal of Urology International*.

[81] Sathyanarayana UG, Maruyama R, Padar A (2004). Molecular detection of noninvasive and invasive bladder tumor tissues and exfoliated cells by aberrant promoter methylation of laminin-5 encoding genes. *Cancer Research*.

[82] Yoder BJ, Skacel M, Hedgepeth R (2007). Reflex UroVysion testing of bladder cancer surveillance patients with equivocal or negative urine cytology: a prospective study with focus on the natural history of anticipatory positive findings. *American Journal of Clinical Pathology*.

[83] Yossepowitch O, Herr HW, Donat SM (2007). Use of urinary biomarkers for bladder cancer surveillance: patient perspectives. *Journal of Urology*.

